# Apple Watch Saves an Athlete: Unmasking Early Detection of Arrhythmogenic Cardiomyopathy

**DOI:** 10.7759/cureus.113337

**Published:** 2026-07-25

**Authors:** Aditi Das Shroddha, Ishikaa Mukherjee, Pier D Lambiase

**Affiliations:** 1 Cardiology, Barts Health NHS Trust, London, GBR; 2 Cardiology, Barts and the London School of Medicine and Dentistry, London, GBR; 3 Cardiology, National Institute for Health and Care Research (NIHR) Barts Biomedical Research Centre (BRC) and NIHR University College London Hospitals/University College London Biomedical Research Centre (UCLH/UCL BRC), London, GBR

**Keywords:** apple watch, arrhythmogenic cardiomyopathy, cardiac mri, idiopathic rvot vt, subepicardial fibrosis

## Abstract

Arrhythmogenic cardiomyopathy (ACM), encompassing arrhythmogenic right ventricular cardiomyopathy (ARVC), is an important cause of ventricular arrhythmias and sudden cardiac death in young individuals, particularly athletes. Early diagnosis remains challenging due to subtle or evolving structural and electrocardiographic findings, and the condition may initially mimic benign idiopathic ventricular tachycardia (VT). We report the case of a 33-year-old athletic male who presented with recurrent palpitations initially documented by a consumer wearable device and was managed as presumed idiopathic right ventricular outflow tract VT (RVOT VT). Despite initial endocardial ablation, he experienced recurrent VT. Subsequent multimodality assessment, including cardiac magnetic resonance imaging and electroanatomic mapping, demonstrated subepicardial fibrosis and extensive right ventricular epicardial scar, consistent with an ACM phenotype. Owing to recurrent ventricular arrhythmias and significant scar burden, a subcutaneous implantable cardioverter-defibrillator was implanted for secondary prevention. This case highlights the diagnostic overlap between idiopathic VT, myocarditis, and early ACM, and underscores the importance of ongoing diagnostic vigilance when arrhythmias recur after apparently successful ablation. It also illustrates the emerging role of wearable technology in the early detection of clinically significant ventricular arrhythmias and emphasises the value of longitudinal, multidisciplinary management in young patients with suspected ACM.

## Introduction

Arrhythmogenic right ventricular cardiomyopathy (ARVC), now often included within the broader spectrum of arrhythmogenic cardiomyopathy (ACM), is a rare but clinically important condition characterised by fibrofatty replacement of the ventricular myocardium and a predisposition to ventricular arrhythmias and sudden cardiac death (SCD) [1]. While classically described as a genetic disorder involving desmosomal proteins, an inflammatory or post-myocarditic pathogenesis has increasingly been recognised, reflecting the phenotypic and etiological heterogeneity of the disease [2]. This variability contributes to significant challenges in early recognition and management.

ARVC/ACM is a leading cause of SCD in young individuals and athletes, often presenting with exertional syncope, palpitations, or collapse [3]. Exercise accelerates disease expression and adverse outcomes, making early diagnosis essential [4]. However, diagnosis remains challenging because ventricular structure may be normal, ECG abnormalities are subtle, and arrhythmias can be nonspecific.

A key diagnostic challenge is distinguishing idiopathic right ventricular outflow tract (RVOT) ventricular tachycardia (VT) from ARVC-related arrhythmias. Idiopathic RVOT VT is typically considered benign, occurring in structurally normal hearts, with excellent outcomes following ablation [4]. However, in some patients, what appears to be idiopathic VT may represent an early phenotypic expression of ACM. Misclassification carries significant consequences, as the latter condition confers risks of disease progression and sudden death despite initial ablation success [5]. Careful integration of multimodality imaging, electroanatomic mapping, and longitudinal follow-up is therefore required to refine the diagnosis.

In parallel, the role of wearable technology in cardiovascular care has expanded rapidly. Devices such as the Apple Watch now enable continuous rhythm monitoring and patient-initiated electrocardiogram (ECG) recordings. While primarily marketed for atrial fibrillation detection, these devices have increasingly been recognised to capture ventricular arrhythmias, sometimes prompting lifesaving interventions [6]. In younger populations where arrhythmias may be intermittent and unexpected, wearable technology offers an additional diagnostic tool that can complement traditional Holter monitoring, exercise testing, electrophysiology studies or implantable loop recording.

We report the case of a young athletic male who initially presented with palpitations documented by his smartwatch and was managed for presumed idiopathic RVOT VT. Despite ablation, he experienced recurrent VT, with subsequent imaging and electroanatomic mapping revealing ventricular scar consistent with ACM. The case highlights the diagnostic uncertainty between myocarditis and ARVC in early disease, the importance of considering ACM in young patients with recurrent arrhythmias, and the emerging utility of wearable technology in the evaluation of young patients with suspected inherited cardiomyopathy.

## Case presentation

A 33-year-old athletic male, with a background of mild asthma (managed with intermittent salbutamol use), presented with recurrent palpitations. He had a history of regular football participation and high-intensity exercise, including weight training at least five times per week. He had no history of smoking, alcohol or recreational drug use, and no family history of cardiomyopathy or SCD.

His cardiology history was notable for an initial presentation approximately two months before the index presentation at Homerton Hospital with exertional palpitations during football. At that time, he was started on bisoprolol and referred to a cardiologist. His first symptomatic episode occurred on Day 0. On a Tuesday evening, he experienced a 12-minute episode of palpitations, followed the next morning by a similar eight-minute episode. In the early hours of Thursday, at 3:00 am, he awoke with frequent ectopic beats noted on his smartwatch single-lead ECG, which progressed to a broad complex tachycardia. This culminated in a sustained 40-minute episode of palpitations, during which he remained haemodynamically stable. The original smartwatch ECG tracings, heart-rate trends, and notification data were unavailable for retrospective review.

**Figure 1:**
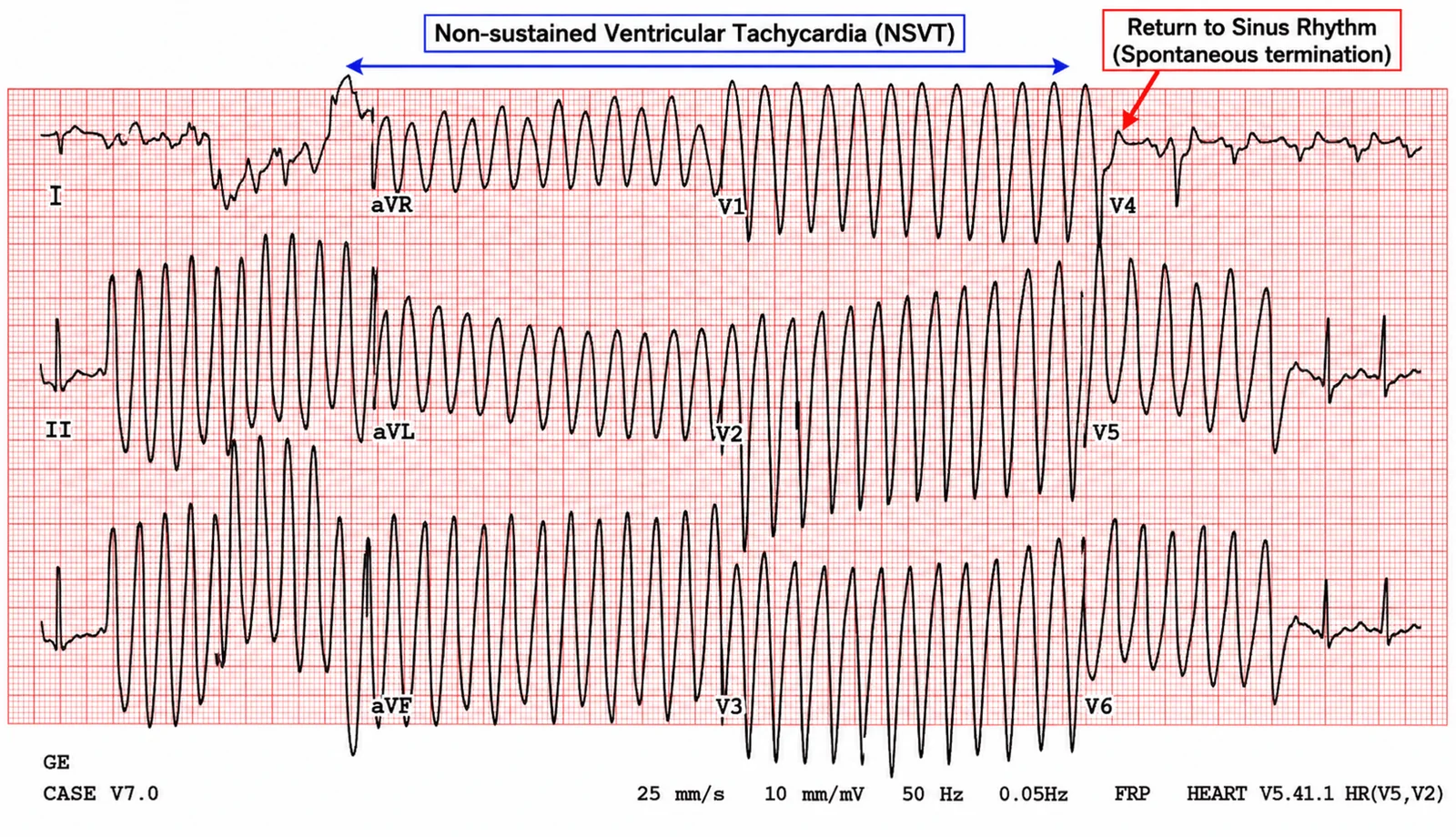
Twelve-lead electrocardiogram obtained on presentation to the emergency department demonstrating sustained monomorphic ventricular tachycardia. Admission 12-lead ECG demonstrating sustained monomorphic ventricular tachycardia with left bundle branch block morphology. The tachyarrhythmia terminates spontaneously (red arrow), followed by restoration of sinus rhythm.

He subsequently self-presented to the emergency department at University College London Hospital. On initial assessment, physical examination was unremarkable, with no clinical signs of heart failure or other abnormal cardiovascular findings. Electrocardiography demonstrated VT with a left bundle branch block morphology and transition in lead V5 (Figure 1). He received intravenous adenosine (6 mg followed by 12 mg) without immediate effect; however, the VT terminated spontaneously approximately five minutes later.

He was admitted for further evaluation and transferred to St. Bartholomew’s Hospital. At this stage, the initial differential diagnosis included idiopathic RVOT VT, ACM, and post-inflammatory cardiomyopathy (prior myocarditis).

Transthoracic echocardiography demonstrated mildly dilated left ventricular and right ventricular cavities with preserved biventricular systolic function (left ventricular ejection fraction (LVEF) 61%, right ventricular fractional area change (RV FAC) 44%, and right ventricular free-wall strain -24%). No regional wall motion abnormalities were identified. Mild biatrial dilatation and mild tricuspid regurgitation were also present, with an estimated right ventricular systolic pressure (RVSP) of 31 mmHg.

**Figure 2:**
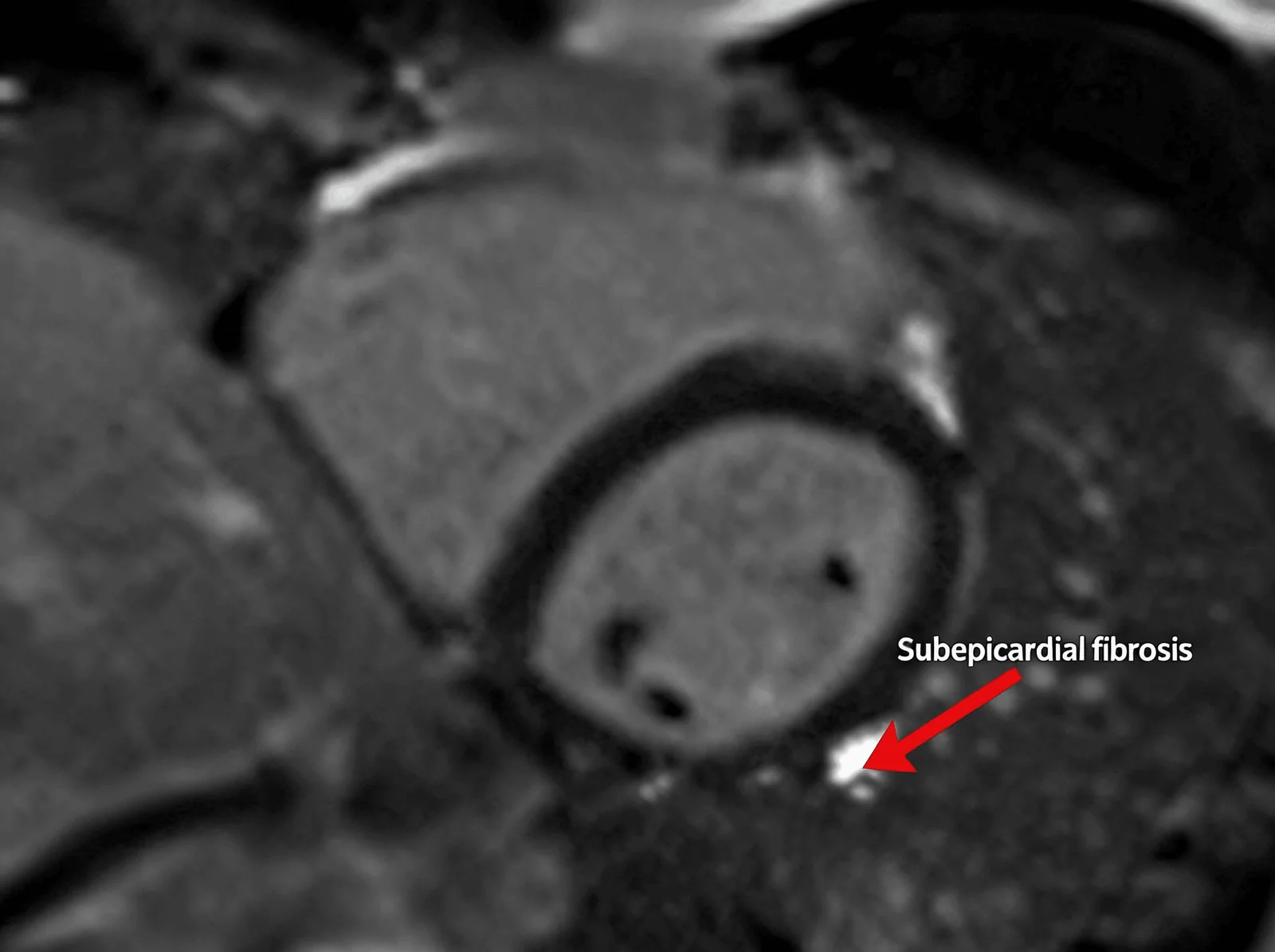
Cardiac magnetic resonance imaging demonstrating subepicardial fibrosis of the basal inferolateral wall. Late gadolinium enhancement cardiac magnetic resonance imaging demonstrating focal subepicardial fibrosis in the basal inferolateral left ventricular wall (red arrow). The non-ischaemic enhancement pattern is consistent with prior myocarditis and may represent a substrate for ventricular arrhythmogenesis.

Cardiac magnetic resonance imaging demonstrated focal subepicardial late gadolinium enhancement (LGE) in the basal inferolateral wall of the left ventricle, consistent with prior myocarditis (Figure 2).

On Day 2, he underwent electrophysiological study with endocardial RVOT ablation for VT. The VT could be sustained for mapping with continuous isoprenaline infusion (1-2 mcg/min) without haemodynamic compromise. There was evidence of epicardial breakthrough on the initial study. A resting ECG post-ablation demonstrated T-wave inversions in leads V1-V2. He remained haemodynamically stable throughout both electrophysiological procedures and subsequent hospital admissions.

Following the initial endocardial ablation, inpatient telemetry over the subsequent days demonstrated recurrent non-sustained ventricular tachycardia (NSVT) episodes, the longest lasting 17 beats. In view of recurrent NSVT, bisoprolol was discontinued and metoprolol 25 mg three times daily was commenced for arrhythmia suppression, with a subsequent reduction in arrhythmic burden. Although the arrhythmic burden gradually decreased, intermittent NSVT persisted. Formal Holter quantification of ventricular ectopic burden was unavailable.

**Figure 3:**
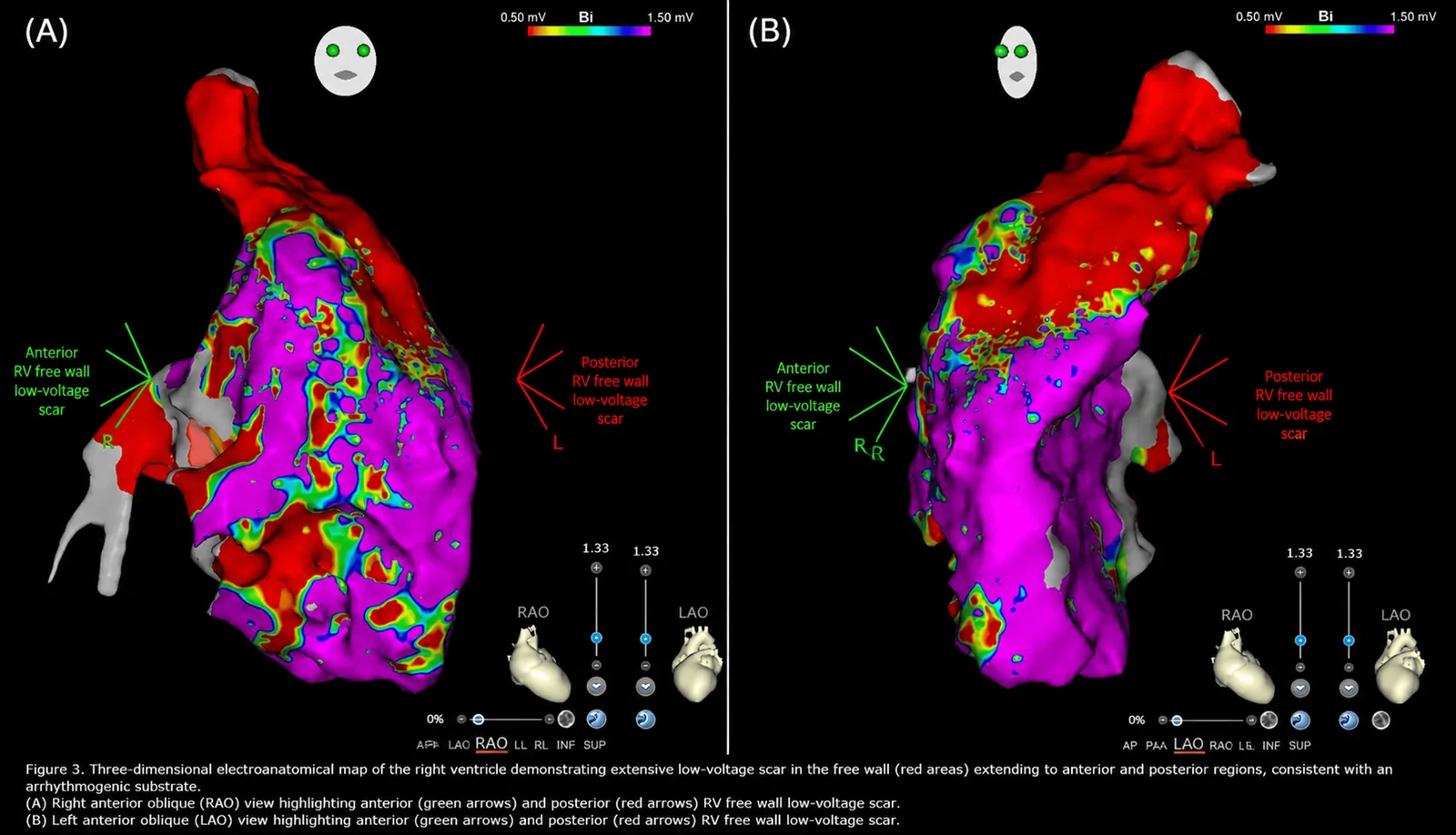
Three-dimensional electroanatomical bipolar voltage mapping demonstrating extensive right ventricular scar substrate. (A) Right anterior oblique (RAO) and (B) left anterior oblique (LAO) views demonstrating extensive low-voltage scar involving the right ventricular free wall. Green arrows identify anterior free-wall scar, while red arrows identify posterior free-wall scar. Purple regions represent preserved myocardium, whereas red regions represent dense scar according to the bipolar voltage scale. The extensive scar burden is consistent with an arrhythmogenic substrate capable of sustaining ventricular tachycardia.

Because of persistent ventricular arrhythmias, flecainide was introduced (50 mg twice daily, increased to 100 mg twice daily) together with nadolol 40 mg once daily. Owing to recurrent ventricular arrhythmias despite the initial endocardial ablation, he subsequently underwent repeat electrophysiological study on Day 11 employing combined endocardial and epicardial mapping, with electroanatomical mapping demonstrating areas of scar affecting the right ventricular free wall and extending to the anterior and posterior aspects of the ventricle (Figure 3). The VT was mapped to the epicardial surface and successfully ablated in this area.

Electroanatomical mapping revealed extensive bipolar low-voltage scar involving the anterior and posterior right ventricular free wall, consistent with a large arrhythmogenic substrate (Figure 3).

Given his recurrent VT and significant scar burden, he underwent implantation of a subcutaneous implantable cardioverter-defibrillator (S-ICD) approximately three months after the index presentation for secondary prevention. Extensive investigations, including CT-PET, viral, Lyme, autoimmune, myositis screening, and cardiomyopathy genetic testing, were negative. No active inflammation was identified, and the absence of a pathogenic variant supported a gene-elusive ACM phenotype, although a post-inflammatory aetiology could not be definitively excluded.

During follow-up over the subsequent 12-18 months, the patient remained clinically well with only occasional ventricular ectopic beats and no sustained ventricular arrhythmias. S-ICD interrogation revealed no therapies delivered. Cardiopulmonary exercise testing demonstrated good functional capacity (240 W workload) with a blunted chronotropic response (peak HR 102 bpm, 55% predicted). Exercise ECG showed only infrequent isolated ventricular extrasystoles without complex ventricular arrhythmias.

At his most recent review (approximately 17 months after the index presentation), he reported ongoing asymptomatic status with stable exercise tolerance. He continued nadolol 20 mg twice daily and flecainide 100 mg twice daily, with ongoing follow-up under electrophysiology and sports cardiology teams.

## Discussion

ARVC, within the broader spectrum of ACM, remains a rare but clinically significant cause of ventricular arrhythmias and SCD in young individuals [1]. The condition has an estimated prevalence between 1:2,000 and 1:5,000, although penetrance is variable and many affected individuals remain undiagnosed until advanced disease or catastrophic presentation [7]. Although registry and autopsy data highlight its contribution to SCD in athletes, initial presentation with sustained VT is uncommon in otherwise healthy, athletic adults [8]. This case therefore illustrates an unusual presentation, initially detected not through conventional clinical pathways but via a consumer wearable device.

Early diagnosis of ACM remains challenging in clinical practice. The 2010 Revised Task Force Criteria provide a structured diagnostic framework based on structural, histological, electrocardiographic, arrhythmic, and genetic parameters, but they demonstrate limited sensitivity in early or non-classical disease presentations [9]. In contrast, the 2020 Padua Criteria retain a six-domain diagnostic framework (morpho-functional, tissue characterisation, repolarisation, depolarisation, arrhythmic, and family/genetic features) while introducing dedicated criteria for left ventricular involvement and placing greater emphasis on LGE detected by cardiac magnetic resonance imaging. These refinements improve recognition of right-dominant, biventricular, and left-dominant phenotypes [10]. In this case, the patient demonstrated only borderline features under the 2010 criteria, including T-wave inversion in V1-V2, subepicardial fibrosis on MRI, and recurrent ventricular arrhythmias, without family history or pathogenic mutation. Under the Padua criteria, the combination of tissue characterisation abnormalities and documented ventricular arrhythmias increased suspicion for ACM; however, definitive classification remained uncertain due to the absence of genetic confirmation and overt structural abnormalities [10]. Given the potential inherited nature of ACM, genetic counselling and cascade family screening should be considered when a pathogenic variant or relevant family history is identified. In this case, negative genetic testing and the absence of a family history reduced the likelihood of an identifiable inherited cause and limited the role of cascade screening.

A key diagnostic challenge is the misclassification of idiopathic RVOT VT in such patients. Idiopathic RVOT VT typically occurs in structurally normal hearts, has a benign prognosis, and is often curable with ablation [11]. However, misdiagnosis can have important consequences, as patients with early ACM may initially respond to ablation only to experience recurrence as the underlying arrhythmogenic substrate progresses. These patients remain at risk of SCD despite initial procedural success [5]. In the present case, recurrent arrhythmia following ablation prompted further evaluation, ultimately revealing myocardial scar consistent with ACM. Under the Padua criteria, the presence of scar detected by electroanatomic mapping together with subepicardial LGE on MRI represents important diagnostic findings that increase suspicion for ACM, while highlighting the limitations of earlier classification systems [10]. This underscores the importance of maintaining diagnostic vigilance in young patients with recurrent ventricular arrhythmias.

The coexistence of subepicardial fibrosis on cardiac MRI and right ventricular scarring on electroanatomic mapping raises important pathophysiological considerations. One possibility is a post-inflammatory cardiomyopathy in which prior myocarditis leads to residual fibrosis that mimics ARVC [3]. Alternatively, myocarditis may act as a disease-modifying or triggering factor in genetically susceptible individuals, contributing to phenotypic expression of ACM [2]. Differentiation between these entities remains challenging, particularly in the absence of genetic confirmation. The Padua criteria recognise the diagnostic importance of LGE patterns, particularly subepicardial or transmural fibrosis in either ventricle, as major structural markers. When combined with electroanatomic evidence of scar, these findings strengthen diagnostic certainty for ACM. Multimodality imaging, including cardiac MRI for tissue characterisation, PET for exclusion of active inflammation, and electroanatomic mapping for electrical scar localisation, is therefore essential in refining diagnostic accuracy.

Risk stratification for SCD in ARVC remains complex. Dedicated risk prediction models have been developed, primarily in cohorts enriched for desmoplakin and plakophilin-2 mutation carriers, incorporating clinical variables such as age, symptoms, electrocardiographic features, premature ventricular contraction burden, and right ventricular ejection fraction [12]. However, caution is required when applying these tools to gene-negative or non-plakophilin variant patients, as risk estimation may be less reliable in these subgroups [13].

Wearable technology represents an emerging adjunct in arrhythmia detection. Consumer devices such as the Apple Watch have primarily been validated for atrial fibrillation detection, but increasing reports demonstrate their ability to capture ventricular arrhythmias [6]. In this case, the smartwatch alerted the patient to an abnormal rhythm and prompted early clinical evaluation. A small number of similar cases have been reported in the literature, supporting the potential role of wearable devices in early arrhythmia detection [14]. While not a replacement for clinical-grade monitoring, such technologies may extend diagnostic reach, particularly in young and active individuals who are less likely to undergo prolonged ambulatory monitoring.

Management in this case further highlights several important principles. Recurrent VT following ablation should prompt reconsideration of the underlying diagnosis and escalation of therapy. In this patient, this led to implantation of an S-ICD for secondary prevention, particularly given the possibility of progressive myocardial fibrosis [15]. This approach is well suited to young patients, avoiding transvenous lead-related complications while providing protection against SCD. Anti-tachycardia pacing is unlikely to be effective in very rapid VT even with transvenous systems, supporting the selected strategy. Modular pacing systems combining subcutaneous defibrillation with leadless pacing may offer future flexibility if pacing support becomes necessary.

Several reports have described patients initially diagnosed with idiopathic VT who were later reclassified as having ACM following disease progression or recurrent arrhythmia [5,10]. This case reflects the same clinical trajectory and emphasises that an initial benign diagnosis should not preclude long-term surveillance. Wearable technology played a key role in early arrhythmia detection, consistent with emerging case literature [6,14].

Exercise plays an important role in disease expression and progression in ACM. High-intensity physical activity has been associated with accelerated phenotypic progression and increased arrhythmic risk [16]. However, exercise restriction may carry significant psychological and social consequences, particularly in athletes. In this case, multidisciplinary input from sports cardiology enabled an individualised approach that balanced risk reduction with preservation of quality of life. This highlights the importance of shared decision-making that integrates clinical risk with patients' lifestyles, identities, and psychosocial well-being.

In summary, this case highlights three key messages. First, VT in young patients should not be assumed to be benign without comprehensive evaluation, particularly in athletic individuals with recurrent arrhythmias. Second, wearable devices may facilitate early rhythm recognition but require confirmation with conventional diagnostic investigations. Finally, optimal management requires a multidisciplinary approach integrating electrophysiology, imaging, sports cardiology, and shared decision-making to deliver individualised long-term care.

## Conclusions

This case outlines the diagnostic complexity encountered in young patients presenting with VT. The initial impression of idiopathic RVOT VT was challenged by recurrent arrhythmias, evolving imaging findings, and a scar identified on electroanatomic mapping, ultimately supporting a diagnosis within the spectrum of ACM. The overlap between idiopathic VT, myocarditis, and early ACM underscores the need for caution when labelling ventricular arrhythmias as benign, particularly in athletic individuals.

Importantly, this case illustrates the emerging role of wearable technology in early arrhythmia detection, providing a timely clinical clue that may otherwise have been missed. However, smartwatch ECGs cannot diagnose structural heart disease and require confirmation with conventional investigations. Multimodality imaging and electrophysiological assessment were essential in refining the diagnosis and guiding management. Long-term multidisciplinary follow-up is essential to balance arrhythmic risk with quality of life. Ultimately, the case emphasises the importance of diagnostic vigilance and comprehensive multidisciplinary care in young athletes presenting with ventricular arrhythmias.
